# Effects of *Phaffia rhodozyma* on microbial community dynamics and tobacco quality during tobacco fermentation

**DOI:** 10.3389/fmicb.2024.1451582

**Published:** 2024-09-17

**Authors:** Jing Mai, Ming-Jun Zhu, Bin-Bin Hu, Hong Zhang, Zhong-Hua Liu, Jian-Feng Sun, Yang Hu, Lu Zhao

**Affiliations:** ^1^Yunnan Academy of Tobacco Agricultural Sciences, Kunming, China; ^2^School of Biology and Biological Engineering, Guangdong Key Laboratory of Fermentation and Enzyme Engineering, Guangzhou Higher Education Mega Center, South China University of Technology, Guangzhou, China; ^3^Yunnan Tobacco Monopoly Bureau, Kunming, China; ^4^Chuxiong State Tobacco Monopoly Bureau, Chuxiong, China

**Keywords:** carotenoids, microbial community, metabolomics, tobacco fermentation, aroma component

## Abstract

**Introduction:**

Carotenoids are important precursors of various aroma components in tobacco and play an important role in the sensory quality of tobacco. *Phaffia rhodozyma* is a species of *Xanthophyllomyces* capable of synthesizing a highly valuable carotenoid-astaxanthin, but has not yet been used in improving tobacco quality.

**Methods:**

The dynamic changes of microbial community and metabolites during tobacco fermentation were analyzed in combination with microbiome and metabolome, and the quality of tobacco after fermentation was evaluated by sensory scores.

**Results:**

*P. rhodozyma* could grow and produce carotenoids in tobacco extract, with a maximum biomass of 6.50 g/L and a maximum carotenoid production of 36.13 mg/L at 100 g/L tobacco extract. Meanwhile, the correlation analysis combined with microbiome and metabolomics showed that *P. rhodozyma* was significantly positively correlated with 11 metabolites such as 6-hydroxyluteolin and quercetin. Furthermore, the contents of alcohols, ketones and esters, which were important aromatic components in fermented tobacco, reached 77.57 μg/g, 58.28 μg/g and 73.51 μg/g, increasing 37.39%, 265.39% and 266.27% compared to the control group, respectively. Therefore, the aroma and flavor, and taste scores of fermented tobacco increased by 0.5 and 1.0 points respectively.

**Discussion:**

This study confirmed that *P. rhodozyma* fermentation could effectively improve the sensory evaluation of tobacco, and provided a novel microbial fermentation method to improve tobacco quality.

## 1 Introduction

Tobacco is an important cash crop in China, and its annual tobacco output and cigarette product output rank among the top in the world (Fang et al., 2017). The quality of tobacco directly affects its economic benefits, and further affects the state of economic income, so the quality of tobacco plays a vital role in the development of the tobacco industry. Unaged flue-cured tobacco is inadequately directly used to make cigarette products due to its strong irritation, insufficient aroma, coarse smoke flavor and heavy miscellaneous gases (Huang et al., 2010; Wang F. et al., 2018; Wang H. et al., 2018). However, normal aging usually takes one to 2 years, during which time is necessary to prevent mildew and insect infestation caused by the environment (Liu et al., 2021).

There are two main sources of microorganisms used in fermentation to improve the quality of tobacco. A kind of microorganisms that was isolated from tobacco could degrade the macromolecular substances such as starch, cellulose and protein in tobacco leaves, improving the smoking quality of tobacco leaves. Dai et al. (2020) isolated a strain of *Bacillus subtilis* ZIM 3 from aged flue-cured tobacco leaves with high amylase activity and cellulase activity, as well as high universality to temperature and pH, which increased the biodegradation efficiency of starch and cellulose in tobacco leaves by 30–48%. Zhang et al. (2024) screened and identified three strains with high amylase, cellulase and protease activities in tobacco, namely *Bacillus cereus* A1, *Bacillus velezensis* A2 and *Bacillus endophyticus* A4. Among them, the co-fermentation of A2 and A4 increased the aroma component content by 108.33%, and significantly improved the sensory quality of heat-not-burn cigarettes. Wen et al. (2023) selected *Bacillus amyloliticus*, *Bacillus kochii* and *Filobasidium magnum* from low-grade tobacco by flow cytometry. The use of these strains in tobacco fermentation improves the aroma composition and sensory evaluation of tobacco, resulting in the reduction of irritation and an improvement in quality of final tobacco product. On the other hand, strains that have been identified were inoculated to tobacco for fermentation. For example, Zheng et al. (2022) inoculated two exogenous microorganisms into cigar tobacco leaves, thereby improving the quality and flavor of cigar tobacco leaves. Song et al. (2024) inoculated *Bacillus altitudinis* to cigar tobacco leaf to enhance macromolecule transformation and aroma production efficiently, and the total aroma production was increased by 43%. Zhang G. et al. (2023) and Zhang Q. et al. (2023) used a new microbial fermentation medium produced by an edible medicinal fungus *Tremella aurantialba* SCT-F3 (CGMCC No.23831) to increase the content of Maillard reaction products and nicotine degradation products in cigar tobacco leaves, thereby improving its quality. Vigliotta et al. (2007) added a yeast *Debaryomyces hansenii* TOB-Y7 to ferment tobacco, which effectively reduced the accumulation of nitrite and TSNA during tobacco fermentation. Furthermore, Yao et al. (2022) used 9 aroma-producing yeasts to apply in artificially solid-state fermentation of cigar filler leaves, and a total of 52 aroma compounds contributed to the flavor of cigar, making its aroma more intense. These microorganisms not only improve the quality of tobacco but also greatly shorten the fermentation time of tobacco.

Carotenoids are important aroma precursors in tobacco, which are degraded and converted into various aroma-causing components such as *β*-damascone and megastigmatrienone during the ripening, preparation, fermentation and combustion of tobacco leaves (Enzell, 1976; Wahlberg et al., 1977). The degradation products of carotenoids have the characteristics of relatively low threshold of aroma substances, low irritation, good aroma quality, and a high contribution rate to tobacco aroma (Sun et al., 2011). Liu et al. (2010) sprayed β-carotene and lutein on the surface of tobacco leaves for fermentation, and the carotenoid degradation products in tobacco showed an obvious increasing trend after fermentation, which effectively improved the quality of tobacco. Maldonado-Robledo et al. (2003) mixed culture by *Bacillus* sp. and *Geotrichum* sp. produced tobacco aroma compounds from the carotenoid lutein, so as to improve the quality of tobacco. Like many plants, terpenoids are important flavor precursors in flue-cured tobacco, and carotenoids are the most important of them, which are degraded by microorganisms into important flavor and aroma components (Wahlberg et al., 1977). Therefore, carotenoids are closely related to the aroma components of tobacco.

*Phaffia rhodozyma* is a species of *Xanthophyllomyces*, which is capable of synthesizing a highly valuable carotenoid-astaxanthin (Flores-Cotera et al., 2021). Lai et al. (2022) used food waste as medium to produce carotenoids by *P. rhodozyma*, leading to carotenoid production of 129.5 mg/L. Jiang et al. (2017) used Jerusalem artichoke extract as carbon source to produce carotenoids by *P. rhodozyma*, and the maximum output reached 982.50 mg/L. In addition, *P. rhodozyma* could also produce carotenoids in mixed carbon sources such as grape juice, sugar cane juice, corn syrup, and so on Gupta et al. (2022).

In addition to the aroma components, the dynamic changes of microorganisms and the metabolites produced during tobacco fermentation also have important impacts on the quality of tobacco. With the popularity of microbiome and metabolomics, it has become possible to use a variety of methods to explore entire bacterial communities and their metabolites (Wang F. et al., 2018; Wang H. et al., 2018; Zhang G. et al., 2023; Zhang Q. et al. 2023). Ning et al. (2023a) showed that amylase treatment in tobacco caused the succession of microbial communities in tobacco, promoted the formation of aroma compounds, and adjusted the chemical composition of tobacco, among which *Basidiomycota* and *Agaricomycetes* were significantly correlated with the aroma, taste and total score of heat-not-burn cigarettes. Fan et al. (2023) used metabolomics to study the dynamic changes of volatile metabolites in cigar tobacco leaves during fermentation, and the results showed that a total of 613 volatile metabolites were detected during fermentation, and 263 metabolites were significantly different. Furthermore, Li et al. (2020) studied the microbial community and metabolites during tobacco fermentation, and found that the increase of fermentation time would lead to significant changes in microbial communities and metabolites, and the compounds involved in sphingolipid metabolism were closely related to the changes in microbial communities. Therefore, it is necessary to study the changes of tobacco quality during fermentation by microbiome and metabolomics.

In recent years, there has been much research on the fermentation of exogenous microorganisms to improve the quality of tobacco, but research on the application of microorganisms that grow and produce carotenoids in tobacco fermentation is still rarely reported. Our previous experiment showed that *P. rhodozyma* could grow and produce astaxanthin in tobacco extract at a higher level among these carotenoids-producing microorganisms (data not shown). At the same time, the culture temperature of *P. rhodozyma* is 22–25°C, which is consistent with the climate of Yunnan Province in China, and there is no need to control the temperature during fermentation. In the present study, we demonstrated *P. rhodozyma* could grow and produce carotenoids in tobacco, and studied their effects on physicochemical properties and aroma components of tobacco during fermentation. In addition, the dynamic changes of microbial community and metabolites during tobacco fermentation were analyzed in combination with microbiome and metabolome, and the quality of tobacco after fermentation was evaluated by sensory scores.

## 2 Materials and methods

### 2.1 Sample and tobacco extracts

The first-cured tobacco samples KRK26 were collected from Wenshan Prefecture, Yunnan Province, China in 2020. KRK26 was crushed at low temperature and tobacco powder was obtained through a 200-mesh sieve. The tobacco powder was added to sterile water and bathed in constant temperature water at 70°C for 35 min, and the supernatant obtained after suction filtration was the tobacco extracts (Ning et al., 2023a).

### 2.2 Strain and cultivation

*P. rhodozyma* was stored in the School of Biological Science and Engineering of South China University of Technology. The seed medium included (g/L) 10.0 yeast extract, 20.0 tryptone, and 20.0 glucose. pH was adjusted to 6.0. The medium was sterilized at 115°C for 30 min. To demonstrate whether *P. rhodozyma* could be grown and metabolized in tobacco, tobacco extracts of 50 g/L, 80 g/L and 100 g/L were used as fermentation medium. The tobacco extracts were sterilized at 115°C for 30 min. The seeds were cultured at 22°C and 200 rpm until OD_600_ of 1.8–2.1, and then inoculated into the fermentation medium at 10% inoculum.

### 2.3 Tobacco fermentation

*P. rhodozyma* was cultured at 22°C and 200 rpm until OD_600_ of 1.8–2.1. The culture was centrifuged at 8000 g for 10 min to collect the cells. The cells were then re-suspended by adding the same volume of sterile water to obtain the suspension. Finally, 8 mL suspension was evenly sprayed onto 20 g of tobacco powder in a plant tissue culture flask. The tobacco was then fermented at 25°C for 3 and 7 days, respectively (Ning et al., 2023b). Unfermented tobacco was used as the control group. The unfermented tobacco which was not inoculated with *P. rhodozyma* was used as the control. The unfermented tobacco was placed at the same temperature and environment for the same time as the fermented tobacco.

### 2.4 Surface structure characterization and basic chemical composition determination

Before observing the surface structure and determining the chemical composition, tobacco powder was dried to a constant weight in an oven at 105°C. In addition, before SEM observation, the SEM samples were evenly glued to the sample stage with conductive adhesive and then sputtered coating. The surface structure of raw and fermented tobacco was operated at a voltage of 10.0 kV using a Merlin scanning electron microscope (SEM) (Carl Zeiss, German) with the magnification of 5,000×. The total sugar and reducing sugar, starch, protein, nicotine and cellulose in tobacco were determined by using China’s tobacco industry standard methods (YC/T 159–2019), (YC/T 216–2013), (YC/T 249–2008), (YC/T 468–2013) and (YC/T 347–2010), respectively.

### 2.5 Analysis of bacterial and fungal microbial communities

The HiPure Bacterial DNA kit and HiPure Fungal DNA kit (Guangzhou Magen Biotechnology Co., Ltd.) were used to isolate bacterial and fungal DNA, respectively. Bacterial microbial community sequences were executed using primers 341F (5’-CCTAYGGGRBGCASCAG-3′) and 806R (5’-GGACTACNNGGGTATCTAAT-3′) to amplify the V3-V4 region of 16S rDNA. Meanwhile, primers ITS5 (5’-GGAAGTAAAAGTCGTAACAAGG-3′) and ITS2 (5′ -GCTGCGTTCTTCATCGATGC-3′) were selected to amplify ITS rDNA for high-throughput sequencing and fungal microbial community analysis (An et al., 2020; Chen et al., 2020). The qualified PCR products were purified by magnetic beads, quantified by enzyme labeling, and mixed according to the concentration of PCR products in the same amount. After full mixing, the PCR products were detected by 2% agarose gel electrophoresis, and the target bands were recovered by glue recovery kit. The library was constructed using TruSeq^®^ DNA PCR-Free Sample Preparation Kit, and the constructed library was quantified by Qubit and Q-PCR. All the Illumina NovaSeq sequencing raw data were submitted to the National Center for Biotechnology Information (NCBI) as a BioProject submission with accession number PRJNA1125638. Principal component analysis (PCA) was the process of extracting the most important elements and structures from multidimensional data through a series of eigenvalues and eigenvector rankings. Based on the Weighted Unifrac distance and the Unweighted Unifrac distance, the principal coordinate combination with the largest contribution rate was selected for plotting and display.

### 2.6 Metabolomics analysis

After freeze-drying by vacuum freeze-dryer, tobacco powder was crushed using a mixer mill (MM 400, Retsch) with a zirconia bead for 1.5 min at 30 Hz. 50 mg freeze-dried powder was dissolved in 1.2 mL 70% methanol solution. The solution was mixed by 30 s of vortex every 30 min, and the procedure was repeated 6 times. Then the solution was centrifuged at 12,000 rpm for 3 min and the extracts were obtained by filtration of the supernatant.

The extracts were analyzed using a UPLC-ESI-MS/MS system, which was equipped with a UPLC column (1.8 μm, 2.1 mm * 100 mm, Agilent SB-C18). The mobile phase consisted of solvent A, pure water with 0.1% formic acid, and solvent B, acetonitrile with 0.1% formic acid. Sample measurements were performed with a gradient program that employed the starting conditions of 95% A, 5% B. Within 9 min, a linear gradient to 5% A, 95% B was programmed, and a composition of 5% A, 95% B was kept for 1 min. Subsequently, a composition of 95% A, 5.0% B was adjusted within 1.1 min and kept for 2.9 min. The flow velocity was set as 0.35 mL per minute, the column oven was set to 40°C, and the injection volume was 4 μL. The effluent was alternatively connected to an ESI-triple quadrupole-linear ion trap (QTRAP)-MS. The ESI source operation parameters were as follows: source temperature 550°C; ion spray voltage (IS) 5,500 V (positive ion mode)/−4,500 V (negative ion mode); ion source gas I (GSI), gas II(GSII), curtain gas (CUR) were set at 50, 60, and 25 psi, respectively. The collision-activated dissociation(CAD) was high. QQQ scans were acquired as MRM experiments with collision gas (nitrogen) set to medium. DP (declustering potential) and CE (collision energy) for individual MRM transitions were done with further DP and CE optimization. A specific set of MRM transitions was monitored for each period according to the metabolites eluted within this period.

For two-group analysis, differential metabolites were determined by VIP (VIP ≥ 1.0) and absolute Log_2_ FC (|Log_2_ FC| ≥ 1.0) (Ma et al., 2021). VIP values were extracted from OPLS-DA results and generated using R package MetaboAnalystR.

### 2.7 Determination of aroma components

The content of aroma components in tobacco leaves was determined using the GC/MS fingerprint technique. The tobacco leaves were dried at 60°C and crushed to powder through a sieve of 60 mesh. 25.00 g (accurate to 0.01 g) of powder samples were weighed and put into a 1,000 mL round-bottom flask, followed by 20 g NaCl and 400 mL of deionized water, which was shaken well and connected to one end of the simultaneous distillation-extraction (SDE) device (Heat in a 60°C water bath). Then 40 mL of dichloromethane was put into a flat-bottom flask at the other end (Heat in an electric jacket). After pretreatment by distillation, the extract was dried with anhydrous sodium sulfate and then added with 1 mL decanol internal standard. The extract was condensed to 1 mL in a water bath at 40°C by a rotary evaporator and then analyzed by gas chromatography–mass spectrometry (GC/MS) with a 0.45 μm filter membrane in the sample bottle. The GC/MS analysis was performed on an Agilent7890A-5975C system equipped with an HP-5MS analytical column (Agilent 19091S-433, 30 m × 0.25 mm × 0.25 μm). There are 76 kinds of standard samples, and their purity was more than 98%. The oven temperature rose from 60°C to 260°C at 2°C/min for 10 min and then rises to 280°C at 5°C/min. The MS ion source temperature and quadrupole temperature were 230°C, and 150°C, respectively, and mass ranged from 30 to 550 amu. Chemical components detected in GC–MS analysis were identified using NIST98. 5,977 GC–MS MassHunter software was used to analyze the data of GC–MS.

### 2.8 Sensory evaluation process

After fermentation, the tobacco powder was made into heat-not-burn cigarettes according to the technical requirements of Yunnan China Tobacco Industrial Co., Ltd., and the cigarettes were scored by five experts with relevant evaluation qualifications. The evaluation included six items: smoke volume (10 points), aroma and flavor (30 points), physiological intensity (10 points), harmony (10 points), irritant (15 points), and taste (25 points), and the scores were the average of the five experts. Meanwhile, the total sensory quality score was expressed as the sum of the average scores of each item. The trial protocol was approved by the ethics committee of the Kunming University Of Science And Technology (KMUST-MEC-210), and all experiments were performed in accordance with the Declaration of Helsinki and informed consent was obtained from all participants.

### 2.9 Analytical method

*P. rhodozyma* biomass and carotenoids content were determined with reference to a previous study (Lai et al., 2022). Briefly, the culture was taken and washed with deionized water after centrifugation, which was dried to constant weight at 105°C to determine its weight difference. For the determination of carotenoid, 1 mL of fermentation broth was taken and centrifuged in a 2 mL centrifuge tube. The supernatant was resuspended with deionized water and repeated three times. Then 0.6 mL of dimethyl sulfoxide was added to the centrifuge tube, and a water bath at 50°C for 5 min, then 2 mL absolute ethanol was added and placed at room temperature for 10 min, and the procedure was repeated until the pigment was completely extracted. Finally, the content of carotenoids was determined by measuring the absorbance value at 480 nm. The content of reducing sugar in tobacco extract was determined by the DNS method.

### 2.10 Statistical analysis

All results were expressed as means ± standard deviation of three replicates of independent experiments. Statistical analysis of the data in this study was performed using the software SPSS 17.0 (SPSS Inc. Chicago). ANOVA and Duncan’s multiple range tests were applied to determine significant differences. Data with a *p*-value <0.05 was considered a statistically significant difference.

## 3 Results

### 3.1 Growth and metabolism of *P. rhodozyma* in tobacco extract

**Figure 1:**
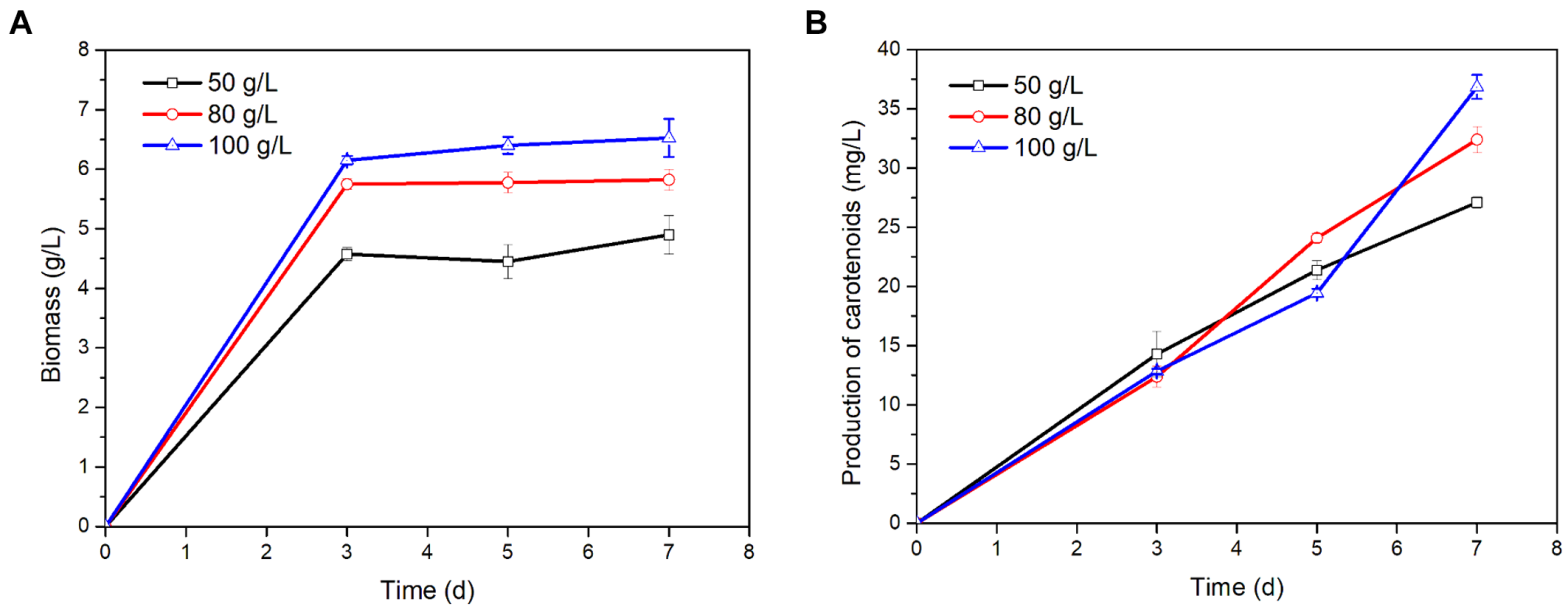
Effects of different concentrations of tobacco extract on *P. rhodozyma* growth **(A)** and carotenoids production **(B)**.

*P. rhodozyma* could grow and produce carotenoids in tobacco extract, and the biomass and carotenoid contents in different concentrations of tobacco extract were significantly different (Figure 1). The biomass of *P. rhodozyma* increased with rising concentration of tobacco extract, reaching a maximum biomass of 6.53 ± 0.32 g/L when the concentration of tobacco extract was 100 g/L. Meanwhile, the production of carotenoids also increased with the extension of fermentation time. When the concentration of tobacco extract was 100 g/L and the fermentation time was 7 days, the maximum carotenoids production reached 36.84 ± 1.02 mg/L. These results showed that *P. rhodozyma* could grow and metabolize in tobacco.

### 3.2 Effect of *P. rhodozyma* on the physicochemical properties of tobacco

#### 3.2.1 Effect of *P. rhodozyma* on surface structure of tobacco

SEM technology was used to observe the changes of tobacco surface structure before and after fermentation. As shown in Supplementary Figure S2, the raw surface structure was intact and there were many uneven and irregular areas. However, there was no significant change in the surface structure of tobacco after fermentation, suggesting that *P. rhodozyma* did not damage the surface structure of tobacco.

#### 3.2.2 Effect of *P. rhodozyma* on basic chemical composition of tobacco

The basic chemical components in tobacco mainly included total sugar, reducing sugar, starch, protein, nicotine and cellulose, which played a non-negligible role in the quality of tobacco. The effects on the basic chemical compositions in tobacco after *P. rhodozyma* fermentation were shown in Supplementary Figure S3. Except for total sugar and reducing sugar, the content of the other basic chemical components had little change after fermentation. The content of the reducing sugar increased significantly during the fermentation process from 26.64 to 31.95% (*p* < 0.01), while the total sugar content decreased significantly from 36.35 to 32.75% (*p* < 0.01).

### 3.3 Effects of *P. rhodozyma* on microbial community diversity and structure in fermented tobacco

#### 3.3.1 Analysis of microbial community diversity

By the termination of culture, samples at different times were collected for α-diversity analysis to imply the effects of *P. rhodozyma* fermentation on community richness and diversity (Supplementary Table S1). The coverage of all samples was 0.99, indicating a high level of confidence in the results. The number of bacterial and fungal OTUs in tobacco decreased with the extension of fermentation time. The OTUs of bacteria decreased from 626 to 460, while the OTUs of fungi decreased from 646 to 584. There was no significant difference in Shannon and Simpson indices in bacteria after fermentation, while both decreased in fungi. In addition, the changes in Chao1 and ACE indices in bacteria and fungi were consistent with those of OTUs.

The Principal Component Analysis (PCA) was used to perform β-diversity analysis of samples to further explore the differences in microbial communities (Supplementary Figure S4). Significant differences in fungal communities between *P. rhodozyma* fermentation and the control group were observed, while no evident differences in bacterial communities. Furthermore, there was no significant change in the microbial communities with the extension of fermentation time.

#### 3.3.2 Analysis of dynamic changes of microbial community structure

**Figure 2:**
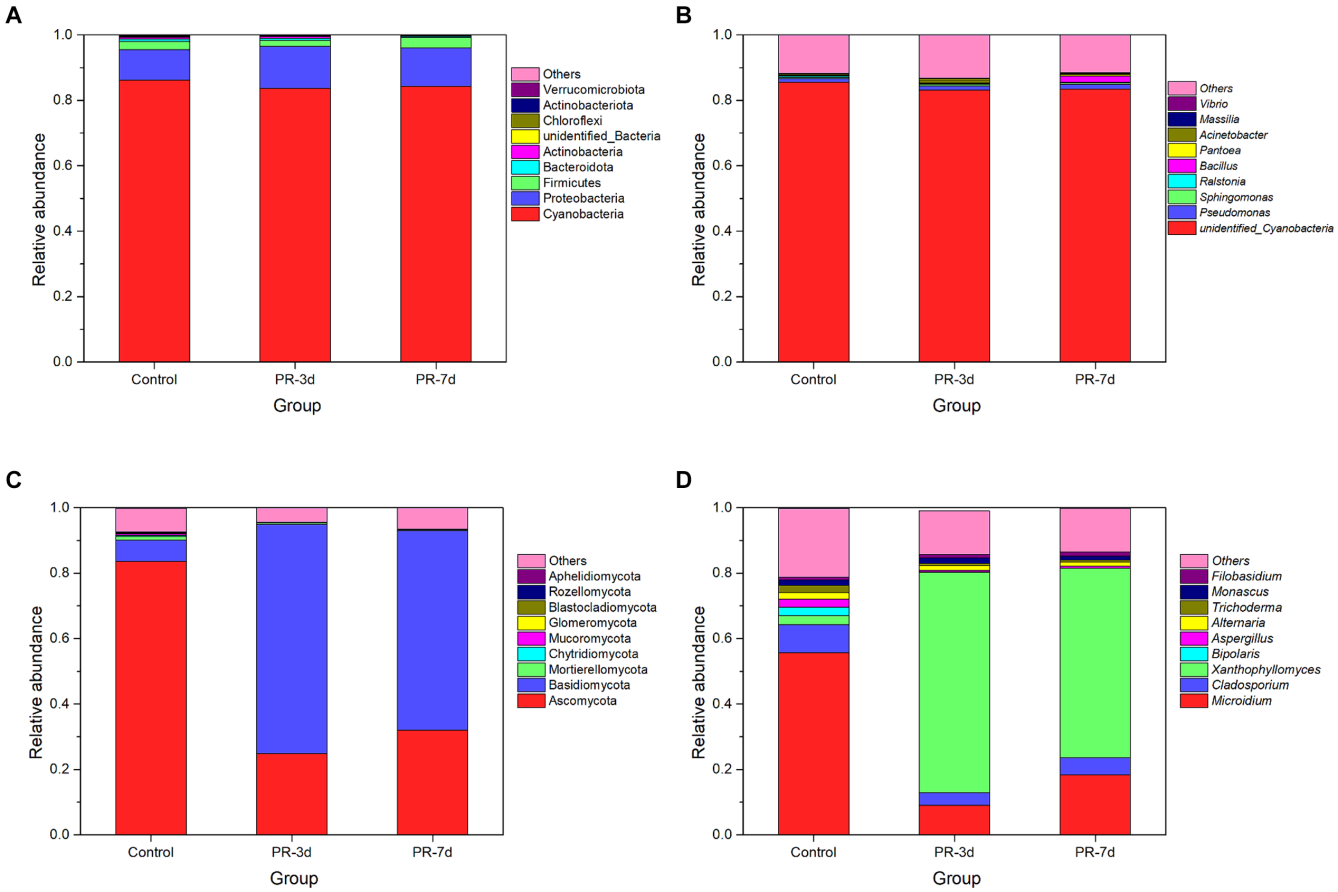
Changes in the relative abundance of bacteria and fungi at phyla and genus levels. **(A)** Phylum level of bacteria. **(B)** Genus level of bacteria. **(C)** Phylum level of fungi, and **(D)** Genus level of fungi.

The relative abundance of bacteria and fungi at different fermentation times was analyzed at the phylum and genus levels (Figure 2). At the level of phylum, the most principal phylum in the initial bacterial community was Cyanobacteria, followed by Proteobacteria and Firmicutes. Ascomycota and Basidiomycota were the dominant phylum in the initial fungal community. After *P. rhodozyma* fermentation, the relative abundance of Cyanobacteria decreased from 86.18 to 83.72% at 3 d and then increased to 84.33% at 7 d, while Proteobacteria increased from 9.29 to 12.78% at 3 d and then decreased to 11.76% at 7 d (Figure 2A). The bacterial community in tobacco did not change significantly after fermentation by *P. rhodozyma*. In contrast, the microbial community of fungi varied greatly, with the relative abundance of Ascomycota decreasing from 83.62 to 24.86% at 3 d and then increasing to 32.06% at 7 d. However, Basidiomycota increased from 6.50 to 70.02% and then decreased to 60.94% (Figure 2C).

Similar to the phylum level, there was no significant change in the microbial community of bacteria at the genus level, while there was a great change in fungi. The bacterial community in tobacco mainly consisted of three genera, *unidentified Cyanobacteria*, *Cladosporium* and *Sphingomonas* (Figure 2B). *Unidentified Cyanobacteria* accounted for the largest proportion with a relative abundance of more than 80% during the fermentation process. It was worth noting that the relative abundance of *Bacillus* increased significantly from 0.30 to 2.00% at 7 d. *Microidium*, *Cladosporium*, and *Xanthophyllomyces* were the most dominant genera of fungi (Figure 2D). The relative abundance of *Microidium* and *Cladosporium* both decreased significantly from 55.59 and 8.66% to 9.03 and 3.85% at 3 d and then increased to 18.27 and 5.29% at 7 d, respectively. In contrast, the relative abundance of *Xanthophyllomyces* increased rapidly from 2.75 to 67.32% at 3 d and then decreased to 57.96% at 7 d. *P. rhodozyma* belonged to the *Xanthophyllomyces*, resulting in its dominance in the fungal community after fermentation. However, the nutrients in tobacco decreased and the inhibitory substances produced by microbial metabolism increased with the fermentation time, which may lead to the decrease in the proportion of *Xanthophyllomyces* at 7 d of fermentation.

### 3.4 Changes of microbial community metabolites in fermented tobacco

**Figure 3:**
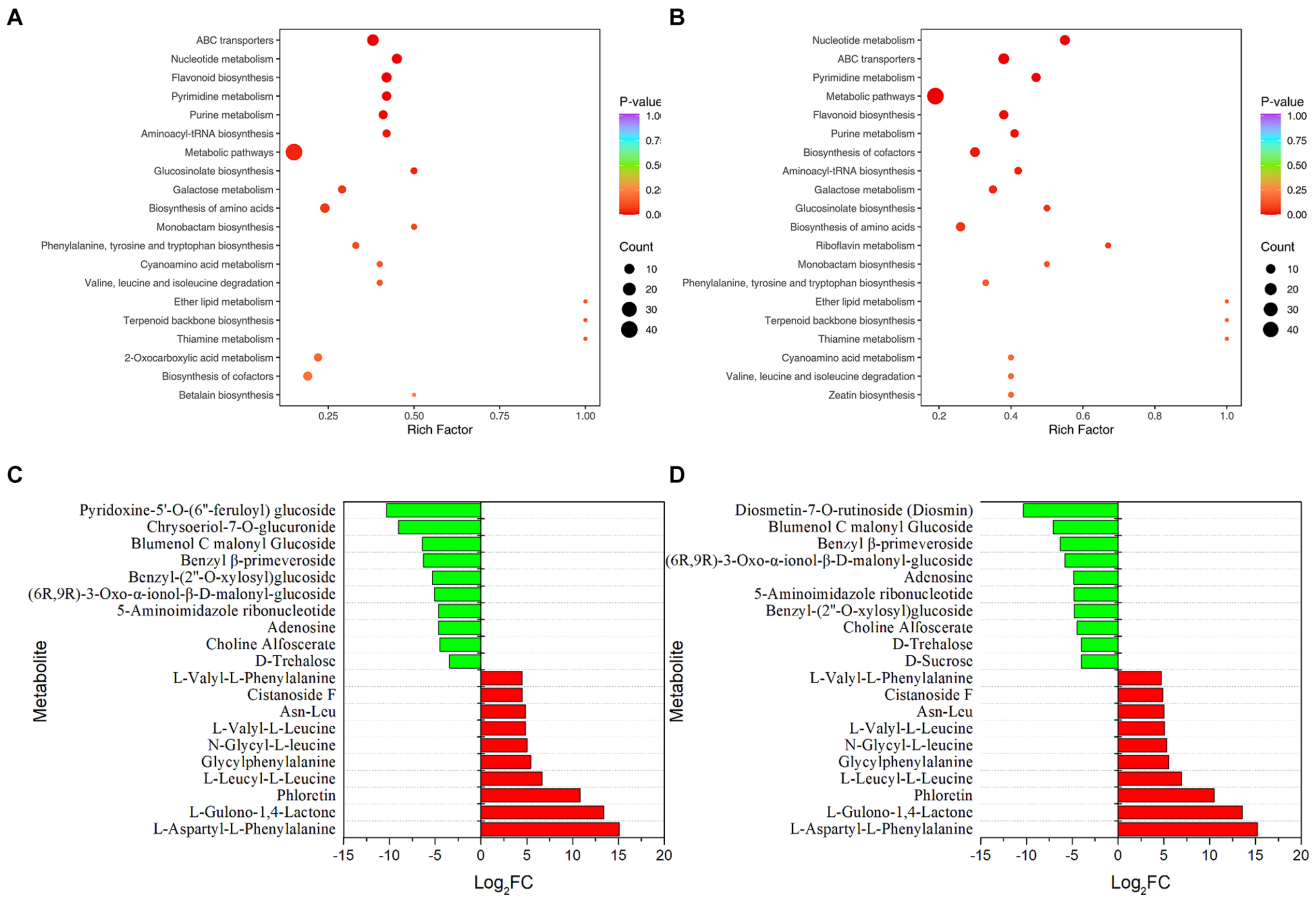
Effects of *P. rhodozyma* fermentation on microbial community metabolites in tobacco. **(A)** KEGG enrichment analysis (3 d). **(B)** KEGG enrichment analysis (7 d). **(C)** Significantly up and down-regulated top10 metabolites (3 d). **(D)** Significantly up and down-regulated top10 metabolites (7 d).

Metabolome analysis was performed to understand the effects of *P. rhodozyma* fermentation on microbial community metabolism in tobacco (Figure 3). The volcano plot was used to show the relative differences in the levels of metabolites between the two groups of samples and the significance of the statistical differences (Supplementary Figure S5). Compared with the control group, a total of 140 and 166 metabolites were significantly different after 3 d and 7 d of fermentation. Wherein, 75 and 91 metabolites were significantly up-regulated and 65 and 75 metabolites were significantly down-regulated for 3 d and 7 d of fermentation, respectively. In addition, only one metabolite was significantly up-regulated and down-regulated between the metabolites of 3 d and 7 d fermentation, indicating that the metabolites of the microbial community had little change with the extension of fermentation time. The metabolites of these microbial communities were mainly divided into the following categories: flavonoids, phenolic acids, lipids, alkaloids, terpenoids, organic acids, amino acids and derivatives, lignans and coumarins, nucleotides and derivatives, quinones and so on. The proportion of flavonoids and phenolic acids were the highest, which were 20.18 and 16.93%, respectively, (Supplementary Figure S6).

The KEGG database was used to annotate the significantly different metabolites, and the annotation results were enriched and analyzed (Figures 3A,B). The closer the *p*-value was to 0, the more significant the enrichment. Compared with the control group, ABC transporters (Ko02010), nucleotide metabolism (Ko01232), flavonoid biosynthesis (Ko00941), pyrimidine metabolism (Ko00240) and purine metabolism (Ko00230) were the top 5 metabolic pathways with *p*-value in the samples after 3 d of fermentation. Except that purine metabolism has been replaced with metabolic pathways (Ko01100), others were the same at 7 d of fermentation as those at 3 d of fermentation. Furthermore, the number of significantly different metabolites in the top 5 metabolic pathways with p-value was more than 5. The top 10 metabolites were significantly up-regulated and down-regulated (Figures 3C,D). The significantly up-regulated top 10 metabolites at 3 and 7 d of fermentation were the same, and the metabolites with the most significant difference were L-aspartyl-L-phenylalanine, followed by L-gulono-1, 4-lactone, and phloretin. In contrast, the significantly down-regulated top 10 metabolites had some differences, among which pyridoxine-5’-*O*-(6″-feruloyl) glucoside, chrysoeriol-7-*O*-glucuronide and blumenol C malonyl glucosidew were the most significant at 3 d, while diosmetin-7-*O*-rutinoside (Diosmin), blumenol C malonyl glucoside and benzyl *β*-primeveroside were the most significant at 7 d. It should be noted that most of the significantly up-regulated top 10 metabolites were amino acids and derivatives, while most of the significantly down-regulated top 10 metabolites were glycosides or sugars.

### 3.5 Combined analysis of microbiome and metabolomics

**Figure 4:**
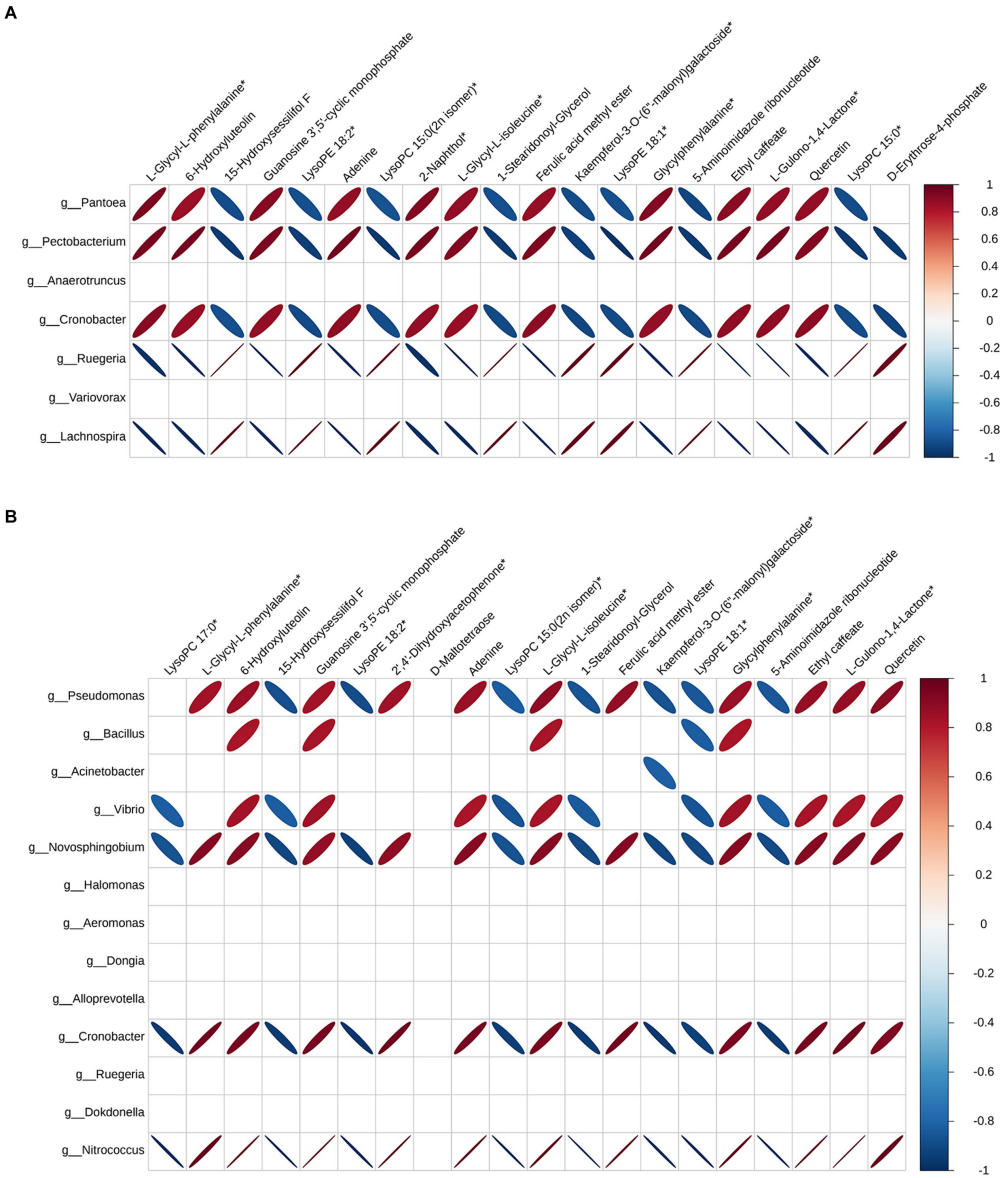
Pearson correlation analysis between differential metabolites and bacteria. **(A)** Fermented 3 d *VS* control and **(B)** Fermented 7 d *VS* control.

**Figure 5:**
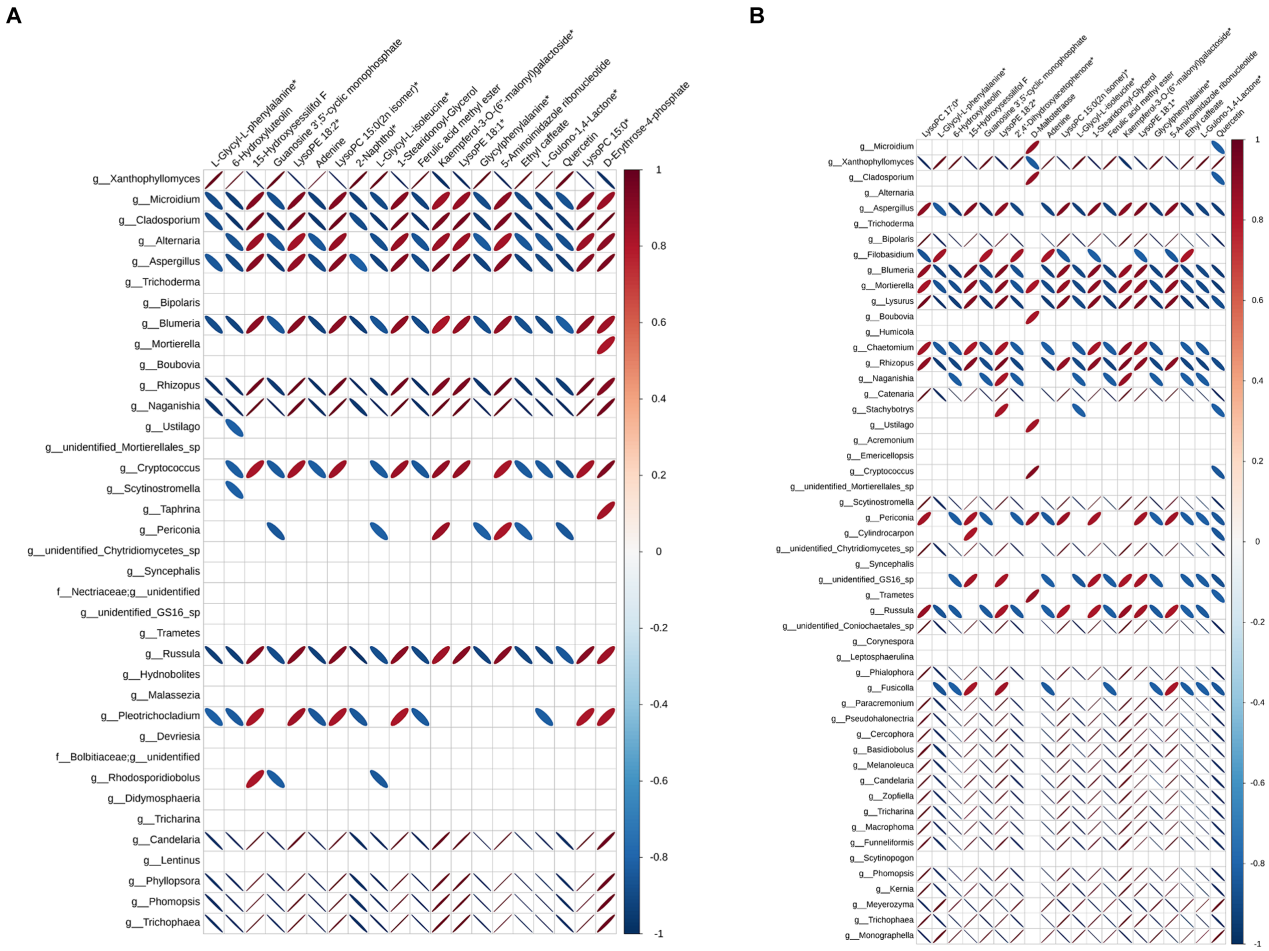
Pearson correlation analysis between differential metabolites and fungi. **(A)** Fermented 3 d *VS* control and **(B)** Fermented 7 d *VS* control.

In order to further analyze the effect of fermentation on tobacco, pearson correlation analysis was performed on differential microorganisms and differential metabolites, and the correlation data of the top 20 differential metabolites and differential microorganisms were extracted to make heat maps. Figure 4 showed the correlation between bacteria and differential metabolites at the genus level, and the correlation between them varies greatly depending on the fermentation time. When the fermentation time was 3 d, *Pantoea*, *Pectobacterium* and *Cronobacter* were positively correlated with 11 differential metabolites, which were L-glycyl-L-phenylalanine, 6-hydroxyluteolin, guanosine 3′,5′-cyclic monophosphate, adenine, 2-naphthol, L-glycyl-L-isoleucine, ferulic acid methyl ester, glycylphenylalanine, ethyl caffeate, L-gulono-1,4-lactone and quercetin. The positively correlated differential metabolites remained largely unchanged, but the associated bacteria became *Pseudomonas*, *Bacilus*, *Vibrio* and *Novosphingobium* at the fermentation time of 7 d. Unlike bacteria, fungi had more genera associated with the top 20 differential metabolites, suggesting that metabolites in the microbial community were mainly produced by fungi (Figure 5). *Xanthophyllomyces* was the most abundant genus of fungi after fermentation, which was significantly related to 11 differential metabolites such as L-glycyl-L-phenylalanine, 6-hydroxyluteolin, guanosine 3′, 5′-cyclic monophosphate, and was the same as the positively correlated metabolites in bacteria. *Microidium* and *Cladosporium*, the second and third most abundant fungal genera in fermentation samples, had the exact opposite differential metabolites with *Xanthophyllomyces* at the fermentation time of 3 d. Nevertheless, *Xanthophyllomyces* dominated the fungal microbial community after fermentation, with a relative abundance of more than 55%, so the metabolites produced in the microbial community were still dominated by *Xanthophyllomyces* related metabolites. However, there was little change in the differential metabolites associated with *Xanthophyllomyces*, while the differential metabolites of *Microidium* and *Cladosporium* were only positively correlated with D-maltotetraose and negatively correlated with quercetin at the fermentation time of 7 d.

### 3.6 Effect of *P. rhodozyma* fermentation on sensory quality of tobacco

#### 3.6.1 Effect of tobacco aroma components after *P. rhodozyma* fermentation

Aroma components were an important index to evaluate the quality of tobacco, among which alcohols, ketones and esters were the main components. As shown in Table 1, the contents of ketones, alcohols and esters in tobacco such as ketones, geranylgeraniol, and dihydroactinidiolide increased to varying degrees after fermentation. The content of dibutyl phthalate increased from 11.77 μg/g to 56.90 μg/g. It was worth noting that the content of cembrenediol 1, 2 and 3, dihydroactinidiolide, 4-hydroxy-*β*-damascone and *β*-damascenone all increased more than twice compared with the control group, and cembrenediol 2 increased by an astonishing 18.14 times. Furthermore, the contents of alcohols, ketones and esters in fermented tobacco were 77.57 μg/g, 58.28 μg/g and 73.51 μg/g, which were increased by 37.39, 265.39 and 266.27% compared with the control group, respectively. These results suggest that *P. rhodozyma* fermentation significantly increased the content of aroma components in tobacco, thereby improving the quality of tobacco. Olfactory activity value (OAV) is an important index to evaluate the quality of tobacco aroma. According to the threshold and attribute descriptions of tobacco aroma components reported in the literature, OAV was calculated for seven of them (Table S3). They all had OVA values greater than 1, with *β*-Damascenone, *β*-Damascone, and megastigmatrienone B having even greater OVA values than 100. These results indicated that these components contribute greatly to the aroma components of fermented tobacco.

**Table 1:** Changes of tobacco aroma components before and after fermentation.

Types	Aroma components	Content (μg/g)	
		Control	Fermentation
Ketones	Solanone	23.20	27.74
	Megastigmatrienone C	7.31	9.35
	Geranyl acetone	7.15	5.05
	Megastigmatrienone B	6.47	8.22
	Megastigmatrienone D	5.68	6.28
	β-Damascenone	3.09	12.96
	Megastigmatrienone A	2.10	2.90
	β-Damascone	0.60	0.86
	4-Hydroxy-β-damascone	0.47	3.50
	Damascone	0.29	0.51
	4-Oxoisophorone	0.10	0.20
Alcohols	Cembrenediol 4	8.32	10.88
	Cembrenediol 3	4.01	11.78
	Cembrenediol 1	1.80	8.58
	Cembrenediol 2	1.38	26.42
	Geranylgeraniol	0.44	0.62
Esters	Dibutyl phthalate	11.77	56.90
	Methyl palmitate	7.55	9.42
	Dihydroactinidiolide	0.75	7.19

#### 3.6.2 Effect of sensory evaluation after *P. rhodozyma* fermentation

The tobacco powder was made into heat-not-burn cigarettes, and then more than 7 experts were organized to smoke the heat-not-burn cigarettes and scored them according to six indicators (Table 2). Through evaluation of experts, the total score of fermented tobacco was 83.0, 1.5 points higher than that of the control group, and 1.0 points and 0.5 points higher in taste, aroma and flavor, respectively. Furthermore, the heat-not-burn cigarettes made from fermented tobacco from *P. rhodozyma* exhibit a good roasted sweetness, elegant smoke and fruity aroma for a better taste.

**Table 2:** Scores of sensory quality.

Groups	Volume of smoke (10–0)	Aroma and flavor (30–0)	Physiological strength (10–0)	Harmony (10–0)	Irritancy (15–0)	Taste (25–0)	Total (100–0)
Control	8.0	23.0	7.0	7.5	13.5	22.5	81.5
Fermentation	8.0	23.5	7.0	7.5	13.5	23.5	83.0

Volume of smoke indicates the concentration of smoke. Aroma and flavor indicates the aromas and tastes felt during the smoking. Physiological strength indicates the physical impact of smoke felt by smokers. Harmony means consistent aroma. Irritation indicates slight and obvious sensory discomfort caused by smoke. Taste refers to the combined sensation of smoking, including comfort, cleanliness, sweetness and so on.

## 4 Discussion

The current studies have shown that the degradation products of carotenoids are important sources of aroma components in tobacco (Popova et al., 2019). Our previous studies have shown that *P. rhodozyma* can grow and produce carotenoids in food waste, Jerusalem artichoke extract or powder, but whether it grows and metabolizes normally in tobacco has not been investigated (Jiang et al., 2017; Lai et al., 2023). The biosynthetic pathway of carotenoids in *P. rhodozyma* consisted of three stages (Supplementary Figure S8). The first stage was the synthesis of the precursor substances Isopentenyl pyrophosphate (IPP) and Dimethylallyl pyrophosphate (DMAPP). In the second stage, IPP was added sequentially based on the precursor material for condensation, and finally phytoene was generated. In the third stage, the phytoene was further structurally modified to produce various carotenoids (Barredo et al., 2017; Gupta et al., 2022). In this process, various terpenes and ketones were produced, which contributed greatly to improving tobacco aroma components. Due to the complexity of the composition in tobacco, it contains not only reducing sugars that provide microbial growth, but also harmful substances such as nicotine (Stedman, 1968). Most substances in tobacco can be extracted by water extraction, thus simulating the environment of tobacco. The results showed that tobacco extract contained a large amount of reducing sugars, which were 7.23 g/L (50 g/L), 11.05 g/L (80 g/L) and 13.24 g/L (100 g/L), respectively (Supplementary Figure S1). This indicated that the higher the concentration of tobacco extract, the higher the reducing sugar content in it. Thus, *P. rhodozyma* could grow and metabolize in tobacco extract with maximum biomass of 4.90 g/L (50 g/L), 5.83 g/L (80 g/L), 6.53 g/L (100 g/L), and maximum carotenoid production of 27.09 g/L (50 g/L), 32.41 g/L (80 g/L) and 36.84 g/L (100 g/L), respectively. These results indicated that *P. rhodozyma* could also grow and metabolize in tobacco.

*P. rhodozyma* fermentation had little effect on the main chemical components in tobacco, only total sugar and reducing sugar had significantly changed. After fermentation, the total sugar content was significantly reduced from 36.35 to 32.75%, which could be consumed by tobacco’s microorganisms and *P. rhodozyma* for growth metabolism. In contrast, the reducing sugar content was significantly increased from 26.64 to 31.30% after fermentation, possibly due to various enzymes secreted by microorganisms such as Bacillus that could degrade polysaccharides in tobacco into reducing sugars. Sun et al. (2023) reported a novel Lytic polysaccharide monooxygenase derived from *Bacillus subtilis* (BsLPMO10A), which exhibited an extensive active-substrate spectrum, particularly for polysaccharides linked via β-1,4 glycosidic bonds, and the co-operation of BsLPMO10A and Glycoside hydrolases released 3,266 ± 182 and 1,725 ± 107 μmol/L of reducing sugars from *Oryza sativa* L. and *Arachis hypogaea* L. straws, respectively. Remarkably, the percentage of *Bacillus* in the genus level also increased significantly after fermentation, corresponding to the result of reducing sugar. *P. rhodozyma* was a species of *Xanthophyllomyces* (Sandmann et al., 2021), and the proportion of *Xanthophyllomyces* in the fungi was significantly increased and dominated the microbial community after fermentation, indicating that it could grow and metabolize in tobacco. In addition, the results of α-diversity analysis suggested that the addition of *P. rhodozyma* reduced the community richness of bacteria and diversity of fungi, indicating that the microbial distribution after fermentation was more concentrated (Huang J. R. et al., 2022).

Metabolome analysis showed that ABC transporters were essential pathways during tobacco fermentation. ABC transporters are widely found in prokaryotes and eukaryotes and are involved in the active transport of a variety of metabolites (Crouzet et al., 2013). After fermentation, sugars such as D-sucrose and lactobiose in ATP transporters were significantly down-regulated, while amino acids such as L-lysine and L-leucine were significantly up-regulated (Supplementary Figure S7). The transport of sugars is closely related to the growth and metabolism of microorganisms (Cirillo, 1961). The significant down-regulation of related sugars in the ABC transporter pathway indicated that they were utilized by microorganisms, which was consistent with the result of the decrease of total sugars in tobacco. Amino acids in tobacco can produce a variety of aroma substances by Maillard reaction with reducing sugars, which is closely related to the production of aroma and taste in tobacco (Geng et al., 2023; Yin et al., 2019). In addition to amino acids, the top 10 metabolites significantly upregulated also included phloretin and cistanoside F. Phloretin is a kind of flavonoid, which has various biological properties such as antioxidase activities, regulation of glucose transportation, and anti-tumor abilities in various diseases (Chang et al., 2012; Sekhon-Loodu et al., 2015). Furthermore, Wang F. et al. (2018) and Wang H. et al. (2018) found that phloretin pretreatment significantly inhibited induced mucin secretion, inflammatory cell infiltration, and inflammatory cytokine release in mouse lungs by cigarette smoke. Cistanche F is a natural active component extracted from cistanche plant, which has effective antioxidant and protective effects on liver injury (Shankar et al., 2018; Sun et al., 2015). These studies showed that *P. rhodozyma* fermented tobacco may reduce the harm of harmful substances to the human body.

To better study how microbial communities affect the quality of tobacco through microbiota metabolism, microbiome and metabolomics were combined analyzed. *Xanthophyllomyces* had the highest proportion in the microbial community, and it was significantly positively correlated with various metabolites such as ethyl caffeate, ferulic acid methyl ester, L-gulono-1,4-lactone, etc. (Bai et al., 2023). Ethyl caffeate is a natural phenolic compound, widely distributed in a variety of plants. Ethyl caffeate has been demonstrated to have many functions such as well antioxidant activity (Könczöl et al., 2012), anti-fibrotic activity (Boselli et al., 2009), and good antidiabetic potential (Wang et al., 2023). Ferulic acid methyl ester, a derivative of ferulic acid, is an effective antibacterial component in plants. Studies have shown that ferulic acid methyl ester exerts antitumor activity and inhibits thrombosis, and has antioxidant and anti-inflammatory functions (Yang et al., 2024). Most of these substances have antioxidant, antibacterial and anti-inflammatory effects, so as to reduce the harm of harmful substances to the human body.

Despite the short fermentation time, the content of aroma components in tobacco still changed evidently. Remarkably, the change of aroma components in tobacco has an important effect on the sensory quality evaluation of tobacco. Based on the results of aroma components, fermentation significantly increased the contents of ketones, alcohols and esters in tobacco aroma components, thereby improving the scores of aroma and flavor and taste in tobacco sensory evaluation. Carotenoid-derived compounds such as damascone and megastigmatrienone occur naturally in tobacco and constitute the basic aroma of tobacco and have an extremely important contribution to the aroma of tobacco (Rodríguez-Bustamante and Sánchez, 2007). On the other hand, the contents of important neutral aroma components such as dibutyl phthalate, cembrenediol, and dihydroactinidiolide were notably increased by 383.43, 271.76 and 858.67%, respectively, which played important roles in determining the flavor of tobacco (Sun et al., 2016; Wang J. A. et al., 2018). The increase in these aromatic components indicated the improvement of fermented tobacco, which was in agreement with the scores of cigarette sensory evaluation experts.

Although *P. rhodozyma* was not capable of degrading macromolecules such as starch and protein like *Bacillus*, nor was it capable of degrading nicotine like *Pseudomonas* (Wen et al., 2023; Zhao et al., 2012), it showed excellent performance in improving the aroma components of fermented tobacco, with the content of alcohols, ketones and esters increasing by 37.39, 265.39 and 266.27%, respectively. Although other microorganisms could also increase the content of aroma components in fermented tobacco, *P. rhodozyma* had better performance compared with the reports (Huang S. et al., 2022; Song et al., 2024; Zhang et al., 2024).

## 5 Conclusion

In summary, this study confirmed that *P. rhodozyma* fermentation could effectively improve the quality of tobacco. The addition of *P. rhodozyma* altered the tobacco microbial community, making it dominant in fermented tobacco. *P. rhodozyma* could produce a variety of aroma substances, and increased the contents of alcohols, ketones and esters in aroma components, thus improving the smoking quality of tobacco and enhancing its sensory score. Most of the microorganisms currently studied in tobacco fermentation are bacteria, and this study mainly focuses on aromatic yeast, which provides new ideas for tobacco fermentation and effectively improves tobacco quality. However, the microbial fermentation technology of tobacco is still in the exploratory stage. Furthermore, the culture temperature of *P. rhodozyma* was 22–25°C, which may only be suitable for large-scale tobacco fermentation in a few places with suitable climates.

## Data Availability

The datasets presented in this study can be found in online repositories. The names of the repository/repositories and accession number(s) can be found at: https://www.ncbi.nlm.nih.gov/, PRJNA1125638.

## References

[ref1] An Q. Cheng J. R. Wang Y. T. Zhu M. J. (2020). Performance and energy recovery of single and two stage biogas production from paper sludge: *Clostridium thermocellum* augmentation and microbial community analysis. Renew. Energy 148, 214–222. doi: 10.1016/j.renene.2019.11.142

[ref2] Bai X. Liu C.-M. Li H.-J. Zhang Z.-P. Cui W.-B. An F.-L.. (2023). Ethyl caffeate attefnuates Aβ-induced toxicity in *Caenorhabditis elegans* AD models via the insulin/insulin-like growth factor-1 signaling pathway. Bioorg. Chem. 139:106714. doi: 10.1016/j.bioorg.2023.106714, PMID: 37454496

[ref3] Barredo J. L. García-Estrada C. Kosalkova K. Barreiro C. (2017). Biosynthesis of Astaxanthin as a Main carotenoid in the Heterobasidiomycetous yeast *Xanthophyllomyces dendrorhous*. J. Fungi. 3:44. doi: 10.3390/jof3030044, PMID: 29371561 PMC5715937

[ref4] Boselli E. Bendia E. Di Lecce G. Benedetti A. Frega N. G. (2009). Ethyl caffeate from *Verdicchio* wine: chromatographic purification and in vivo evaluation of its antifibrotic activity. J. Sep. Sci. 32, 3585–3590. doi: 10.1002/jssc.200900304, PMID: 19813225

[ref5] Chang W.-T. Huang W.-C. Liou C.-J. (2012). Evaluation of the anti-inflammatory effects of phloretin and phlorizin in lipopolysaccharide-stimulated mouse macrophages. Food Chem. 134, 972–979. doi: 10.1016/j.foodchem.2012.03.002, PMID: 23107715

[ref6] Chen Q.-L. Cai L. Wang H.-C. Cai L.-T. Goodwin P. Ma J.. (2020). Fungal composition and diversity of the tobacco leaf phyllosphere during curing of leaves. Front. Microbiol. 11:554051. doi: 10.3389/fmicb.2020.554051, PMID: 33013785 PMC7499341

[ref7] Cirillo V. P. (1961). Sugar transport in microorganisms. Ann. Rev. Microbiol. 15, 197–218. doi: 10.1146/annurev.mi.15.100161.001213

[ref8] Crouzet J. Roland J. Peeters E. Trombik T. Ducos E. Nader J.. (2013). NtPDR1, a plasma membrane ABC transporter from *Nicotiana tabacum*, is involved in diterpene transport. Plant Mol. Biol. 82, 181–192. doi: 10.1007/s11103-013-0053-0, PMID: 23564360

[ref9] Dai J. C. Dong A. J. Xiong G. X. Liu Y. Q. Hossain M. S. Liu S. Y.. (2020). Production of highly active extracellular amylase and cellulase from *Bacillus subtilis ZIM3* and a recombinant strain with a potential application in tobacco fermentation. Front. Microbiol. 11:01539. doi: 10.3389/fmicb.2020.01539, PMID: 32793132 PMC7385192

[ref10] Enzell C. F. (1976). Terpenoid components of leaf and their relationship to smoking quality and aroma. Recent Adv. Tobacco Sci. 2, 32–60. doi: 10.13140/2.1.1028.5128

[ref11] Fan J. Kong G. Yao H. Wu Y. Zhao G. Li F.. (2023). Widely targeted metabolomic analysis reveals that volatile metabolites in cigar tobacco leaves dynamically change during fermentation. Biochem. Biophys. Rep. 35:101532. doi: 10.1016/j.bbrep.2023.101532, PMID: 37637940 PMC10457684

[ref12] Fang J. Lee K. Sejpal N. (2017). The China National Tobacco Corporation: from domestic to global dragon? Glob. Public Health 12, 315–334. doi: 10.1080/17441692.2016.1241293, PMID: 27737622 PMC5553430

[ref13] Flores-Cotera L. B. Chávez-Cabrera C. Martínez-Cárdenas A. Sánchez S. García-Flores O. U. (2021). Deciphering the mechanism by which the yeast *Phaffia rhodozyma* responds adaptively to environmental, nutritional, and genetic cues. J. Ind. Microbiol. Biotechnol. 48, 9–10. doi: 10.1093/jimb/kuab048 PMC8788774 34302341

[ref14] Geng Z. He P. Gao H. Liu J. Qiu J. Cai B. (2023). Aroma precursors of cigars from different tobacco parts and origins, and their correlations with sensory characteristics. Front. Plant Sci. 14:1264739. doi: 10.3389/fpls.2023.1264739, PMID: 38192690 PMC10773810

[ref15] Gupta I. Adin S. N. Panda B. P. Mujeeb M. (2022). *β*-Carotene-production methods, biosynthesis from *Phaffia rhodozyma*, factors affecting its production during fermentation, pharmacological properties: A review. Biotechnol. Appl. Biochem. 69, 2517–2529. doi: 10.1002/bab.2301, PMID: 35048411

[ref16] Huang J.-R. Chen X. Hu B.-B. Cheng J.-R. Zhu M.-J. (2022). Bioaugmentation combined with biochar to enhance thermophilic hydrogen production from sugarcane bagasse. Bioresour. Technol. 348:126790. doi: 10.1016/j.biortech.2022.126790, PMID: 35104653

[ref17] Huang S. Liu D. Chen M. Xi G. Yang P. Jia C.. (2022). Effects of *Bacillus subtilis* subsp. on the microbial community and aroma components of flue-cured tobacco leaves based on metagenome analysis. Arch. Microbiol. 204:726. doi: 10.1007/s00203-022-03347-1, PMID: 36427112

[ref18] Huang J. Yang J. Duan Y. Gu W. Gong X. Zhe W.. (2010). Bacterial diversities on unaged and aging flue-cured tobacco leaves estimated by 16S rRNA sequence analysis. Appl. Microbiol. Biotechnol. 88, 553–562. doi: 10.1007/s00253-010-2763-4, PMID: 20645083

[ref19] Jiang G. L. Zhou L. Y. Wang Y. T. Zhu M. J. (2017). Astaxanthin from Jerusalem artichoke: production by fed-batch fermentation using *Phaffia rhodozyma* and application in cosmetics. Process Biochem. 63, 16–25. doi: 10.1016/j.procbio.2017.08.013

[ref20] Könczöl Á. Béni Z. Sipos M. M. Rill A. Háda V. Hohmann J.. (2012). Antioxidant activity-guided phytochemical investigation of *Artemisia gmelinii* Webb. Ex Stechm.: isolation and spectroscopic challenges of 3,5-*O*-dicaffeoyl (epi?) quinic acid and its ethyl ester. J. Pharm. Biomed. Anal. 59, 83–89. doi: 10.1016/j.jpba.2011.10.012, PMID: 22079045

[ref21] Lai J.-X. Chen X. Bu J. Hu B.-B. Zhu M.-J. (2022). Direct production of astaxanthin from food waste by *Phaffia rhodozyma*. Process Biochem. 113, 224–233. doi: 10.1016/j.procbio.2022.01.003 37278155

[ref22] Lai J. X. Liu W. P. Bu J. Chen X. Hu B. B. Zhu M. J. (2023). Enhancement of astaxanthin production from food waste by *Phaffia rhodozyma* screened by flow cytometry and feed application potential. Biotechnol. Appl. Biochem. 70, 1817–1829. doi: 10.1002/bab.2484 37278155

[ref23] Li J. J. Zhao Y. Y. Qin Y. Q. Shi H. Z. (2020). Influence of microbiota and metabolites on the quality of tobacco during fermentation. BMC Microbiol. 20:356. doi: 10.1186/s12866-020-02035-8, PMID: 33213368 PMC7678276

[ref24] Liu J. Li Y. Huang F. Ji X. Zhao M. J. J. (2010). Influence of spraying carotenoids on flue-cured tobacco quality before fermentation. J. Jilin Agric. Univ. 32, 606–611. doi: 10.13327/j.jjlau.2010.06.013

[ref25] Liu F. Wu Z. Y. Zhang X. P. Xi G. L. Zhao Z. Lai M.. (2021). Microbial community and metabolic function analysis of cigar tobacco leaves during fermentation. Microbiology 10:e1171. doi: 10.1002/mbo3.1171, PMID: 33970539 PMC8483401

[ref26] Ma Y. Ling T.-J. Su X.-Q. Jiang B. Nian B. Chen L.-J.. (2021). Integrated proteomics and metabolomics analysis of tea leaves fermented by *aspergillus Niger*, *aspergillus tamarii* and *Aspergillus fumigatus*. Food Chem. 334:127560. doi: 10.1016/j.foodchem.2020.127560, PMID: 32711271

[ref27] Maldonado-Robledo G. Rodriguez-Bustamante E. Sanchez-Contreras A. Rodriguez-Sanoja R. Sanchez S. (2003). Production of tobacco aroma from lutein. Specific role of the microorganisms involved in the process. Appl. Microbiol. Biotechnol. 62, 484–488. doi: 10.1007/s00253-003-1315-6 12827317

[ref28] Ning Y. Mai J. Hu B.-B. Lin Z.-L. Chen Y. Jiang Y.-L.. (2023a). Study on the effect of enzymatic treatment of tobacco on HnB cigarettes and microbial succession during fermentation. Appl. Microbiol. Biotechnol. 107, 4217–4232. doi: 10.1007/s00253-023-12577-2 37209161

[ref29] Ning Y. Zhang L.-Y. Mai J. Su J.-E. Cai J.-Y. Chen Y.. (2023b). Tobacco microbial screening and application in improving the quality of tobacco in different physical states. Bioresour. Bioprocess. 10:32. doi: 10.1186/s40643-023-00651-6, PMID: 38647749 PMC10992236

[ref30] Popova V. Ivanova T. Prokopov T. Nikolova M. Stoyanova A. Zheljazkov V. D. J. M. (2019). Carotenoid-related volatile compounds of tobacco (*Nicotiana tabacum L.*) essential oils. Molecules 24:446. doi: 10.3390/molecules24193446, PMID: 31547525 PMC6804150

[ref31] Rodríguez-Bustamante E. Sánchez S. (2007). Microbial production of C_13_-norisoprenoids and other aroma compounds via carotenoid cleavage. Crit. Rev. Microbiol. 33, 211–230. doi: 10.1080/10408410701473306, PMID: 17653988

[ref32] Sandmann G. Pollmann H. Gassel S. Breitenbach J. (2021). *Xanthophyllomyces dendrorhous*, a versatile platform for the production of carotenoids and other acetyl-CoA-derived compounds. Adv. Exp. Med. Biol. 1261, 137–151. doi: 10.1007/978-981-15-7360-6_11, PMID: 33783736

[ref33] Sekhon-Loodu S. Ziaullah S. Rupasinghe H. P. V. (2015). Docosahexaenoic acid ester of phloridzin inhibit lipopolysaccharide-induced inflammation in THP-1 differentiated macrophages. Int. Immunopharmacol. 25, 199–206. doi: 10.1016/j.intimp.2015.01.019, PMID: 25637769

[ref34] Shankar G. Borkar R. M. Udutha S. Anagoni S. P. Srinivas R. (2018). Identification and structural characterization of in vivo metabolites of balofloxacin in rat plasma, urine and feces samples using Q-TOF/LC/ESI/MS/MS: in silico toxicity studies. J. Pharm. Biomed. Anal. 159, 200–211. doi: 10.1016/j.jpba.2018.06.050, PMID: 29990887

[ref35] Song W. Chen X. Yu J. Qiao J. Yang J. Chen X.. (2024). Effects of *Bacillus altitudinis* inoculants on cigar tobacco leaf fermentation. Front. Bioeng. Biotechnol. 12:1417601. doi: 10.3389/fbioe.2024.1417601, PMID: 39045536 PMC11264575

[ref36] Stedman R. L. (1968). Chemical composition of tobacco and tobacco smoke. Chem. Rev. 68, 153–207. doi: 10.1021/cr60252a002 4868017

[ref37] Sun X.-B. Gao D.-Y. Cao J.-W. Liu Y. Rong Z.-T. Wang J.-K.. (2023). BsLPMO10A from *Bacillus subtilis* boosts the depolymerization of diverse polysaccharides linked via *β*-1,4-glycosidic bonds. Int. J. Biol. Macromol. 230:123133. doi: 10.1016/j.ijbiomac.2023.123133 36621733

[ref38] Sun J. G. He J. W. Wu F. G. Tu S. X. Yan T. J. Si H.. (2011). Comparative analysis on chemical components and sensory quality of aging flue-cured tobacco from four main tobacco areas of China. Agric. Sci. China 10, 1222–1231. doi: 10.1016/S1671-2927(11)60113-2

[ref39] Sun X. D. Li Y. Y. Wang L.-J. Wu D. Zheng S.-J. Wang W.-M.. (2016). Capturing tobacco specific *N*-nitrosamines (TSNA) in industrial tobacco extract solution by ZnO modified activated carbon. Microporous Mesoporous Mater. 222, 160–168. doi: 10.1016/j.micromeso.2015.10.001

[ref40] Sun J. Song Y. Zhang J. Huang Z. Huo H. Zheng J.. (2015). Characterization and quantitative analysis of phenylpropanoid amides in eggplant (*Solanum melongena L.*) by high performance liquid chromatography coupled with diode array detection and hybrid ion trap time-of-flight mass spectrometry. J. Agric. Food Chem. 63, 3426–3436. doi: 10.1021/acs.jafc.5b00023, PMID: 25796999

[ref001] Vigliotta G. Di Giacomo M. Carata E. Massardo D. R. Tredici S. M. Silvestro D.. (2007). Nitrite metabolism in Debaryomyces hansenii TOB-Y7, a yeast strain involved in tobacco fermentation. Appl. Microbiol. Biotechnol. 75, 633–645. doi: 10.1007/s00253-007-0867-2 17318539

[ref41] Wahlberg I. Karlsson K. Austin D. J. Junker N. Roeraade J. Enzell C. R.. (1977). Effects of flue-curing and ageing on the volatile, neutral and acidic constituents of Virginia tobacco. Phytochemistry 16, 1217–1231. doi: 10.1016/S0031-9422(00)94363-2

[ref42] Wang Q. Li N. Wang Y. Li R. Jia Y. Zhou J.. (2023). Studies on the key constituents and the related mechanisms of *Clerodendranthus spicatus* in the treatment of diabetes based on network pharmacology. J. Ethnopharmacol. 303:115949. doi: 10.1016/j.jep.2022.115949, PMID: 36435408

[ref43] Wang J.-A. Yang G.-H. Li C.-X. (2018). Zonal distribution of neutral aroma components in flue-cured tobacco leaves. Phytochem. Lett. 24, 125–130. doi: 10.1016/j.phytol.2018.01.014

[ref44] Wang H. Yang T. Wang T. Hao N. Shen Y. Wu Y.. (2018). Phloretin attenuates mucus hypersecretion and airway inflammation induced by cigarette smoke. Int. Immunopharmacol. 55, 112–119. doi: 10.1016/j.intimp.2017.12.009, PMID: 29245072

[ref45] Wang F. Zhao H. Xiang H. Wu L. Men X. Qi C.. (2018). Species diversity and functional prediction of surface bacterial communities on aging flue-cured tobaccos. Curr. Microbiol. 75, 1306–1315. doi: 10.1007/s00284-018-1525-x, PMID: 29869679

[ref46] Wen C. Zhang Q. Zhu P. Hu W. Jia Y. Yang S.. (2023). High throughput screening of key functional strains based on improving tobacco quality and mixed fermentation. Front. Bioeng. Biotechnol. 11:1108766. doi: 10.3389/fbioe.2023.1108766, PMID: 36714011 PMC9880406

[ref47] Yang Y. Wang S. Liu X. Zhang W. Tong W. Luo H.. (2024). Interactions of ferulic acid and ferulic acid methyl ester with endogenous proteins: determination using the multi-methods. Heliyon 10:e24605. doi: 10.1016/j.heliyon.2024.e24605, PMID: 38312678 PMC10835327

[ref48] Yao L. Huang C. Ding J. Zhang T. Yu J. Yang C.. (2022). Application of yeast in plant-derived aroma formation from cigar filler leaves. Front. Bioeng. Biotechnol. 10:1093755. doi: 10.3389/fbioe.2022.1093755, PMID: 36619396 PMC9815610

[ref49] Yin F. Karangwa E. Song S. Duhoranimana E. Lin S. Cui H.. (2019). Contribution of tobacco composition compounds to characteristic aroma of Chinese faint-scent cigarettes through chromatography analysis and partial least squares regression. J. Chromatogr. B 1105, 217–227. doi: 10.1016/j.jchromb.2018.12.001, PMID: 30611933

[ref50] Zhang L.-Y. Mai J. Shi J.-F. Ai K.-B. He L. Zhu M.-J.. (2024). Study on tobacco quality improvement and bacterial community succession during microbial co-fermentation. Ind. Crop. Prod. 208:117889. doi: 10.1016/j.indcrop.2023.117889

[ref51] Zhang Q. Yang S. Yang Z. Zheng T. Li P. Zhou Q.. (2023). Effects of a novel microbial fermentation medium produced by *Tremella aurantialba* SCT-F3 on cigar filler leaf. Front. Microbiol. 14:1267916. doi: 10.3389/fmicb.2023.1267916, PMID: 37808308 PMC10556473

[ref52] Zhang G. Yao H. Zhao G. Wu Y. Xia H. Li Y.. (2023). Metabolomics reveals the effects producing region and fermentation stage on substance conversion in cigar tobacco leaf. Chem. Biol. Technol. Agric. 10:66. doi: 10.1186/s40538-023-00444-1

[ref53] Zhao L. Zhu C. Gao Y. Wang C. Li X. Shu M.. (2012). Nicotine degradation enhancement by *Pseudomonas stutzeri* ZCJ during aging process of tobacco leaves. World J. Microbiol. Biotechnol. 28, 2077–2086. doi: 10.1007/s11274-012-1010-9, PMID: 22806029

[ref54] Zheng T. F. Zhang Q. Y. Wu Q. Y. Li D. L. Wu X. Y. Li P. H.. (2022). Effects of inoculation with *Acinetobacter* on fermentation of cigar tobacco leaves. Front. Microbiol. 13:911791. doi: 10.3389/fmicb.2022.911791, PMID: 35783443 PMC9248808

