# Supplementary material for: Effects of Phaffia rhodozyma on microbial community dynamics and tobacco quality during tobacco fermentation
Source: Front Microbiol. 2024 Sep 17;15:1451582. doi: 10.3389/fmicb.2024.1451582 (PMC11442207; doi:10.3389/fmicb.2024.1451582)
Supplement: Supplementary file 1 [file Data_Sheet_1.docx]

**Table S1** α-diversity analysis of tobacco microbial community in *P. rhodozyma* fermented tobacco.

(PR-3d:*P. rhodozyma* fermented 3d; PR-7d:*P. rhodozyma* fermented 7d)

| Microorganism | Group | OTUs | Shannon | Simpson | Chao1 | ACE | Coverage |
| --- | --- | --- | --- | --- | --- | --- | --- |
| Bacteria | Control | 626 | 1.54 | 0.27 | 759.44 | 783.93 | 0.99 |
|  | PR-3d | 561 | 1.63 | 0.31 | 747.72 | 757.64 | 0.99 |
|  | PR-7d | 460 | 1.58 | 0.31 | 585.28 | 608.66 | 0.99 |
| Fungi | Control | 646 | 4.41 | 0.85 | 982.35 | 903.46 | 0.99 |
|  | PR-3d | 608 | 2.80 | 0.52 | 821.73 | 862.97 | 0.99 |
|  | PR-7d | 584 | 2.97 | 0.61 | 792.39 | 821.80 | 0.99 |

Table S2 Statistical analysis of tobacco chemical composition changed before and after fermentation

| Chemical composition | Parameter | | |
| --- | --- | --- | --- |
|  | Freedom | F value | P value |
| Total sugar | 1 | 170.73 | < 0.01 |
| Reducing sugar | 1 | 34.97 | < 0.01 |
| Starch | 1 | 0.31 | 0.61 |
| Protein | 1 | 4.51 | 0.10 |
| Nicotine | 1 | 4.05 | 0.11 |
| Cellulose | 1 | 2.29 | 0.21 |

Post-hoc analysis was not possible because there were not enough groups.

Table S3 Changes of tobacco aroma components before and after fermentation.

| Compound | Threshold (μg/g) | OAV | | Description |
| --- | --- | --- | --- | --- |
|  |  | Control | Fermentation |  |
| Solanone | 1.82 | 12.75 | 15.24 | Carrot, tobacco |
| *β*-Damascenone | 2.0×10^-6^ | 1545000.00 | 6480000.00 | Flowers aroma |
| *β*-Damascone | 1.3×10^-6^ | 46153.85 | 66153.85 | Rich rose aroma |
| Damascone | 7.0×10^-3^ | 41.43 | 72.86 | Strong rose, fruity |
| Dihydroactinidiolide | 0.28 | 2.68 | 25.68 | Coumarin-like, musky |
| Geranyl acetone | 0.19 | 37.63 | 26.58 | Fresh, floral, rose, green, fruity, magnolia |
| Megastigmatrienone B | 2.9×10^-3^ | 223.10 | 283.45 | Tobacco type, nutty |





**Figure S1** Reducing sugar content in tobacco extract with different concentrations.


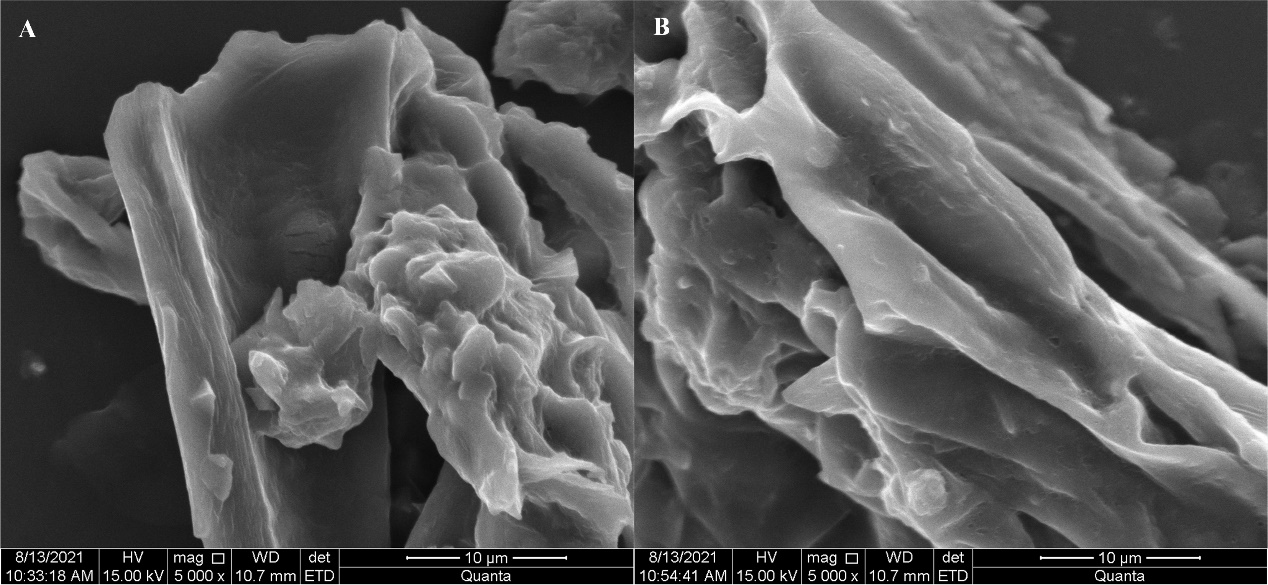


**Figure S2** SEM of raw tobacco (A) and *P. rhodozyma* fermented tobacco (B).





**Figure S3** Effects of *P. rhodozyma* fermentation on the basic chemical components of tobacco.


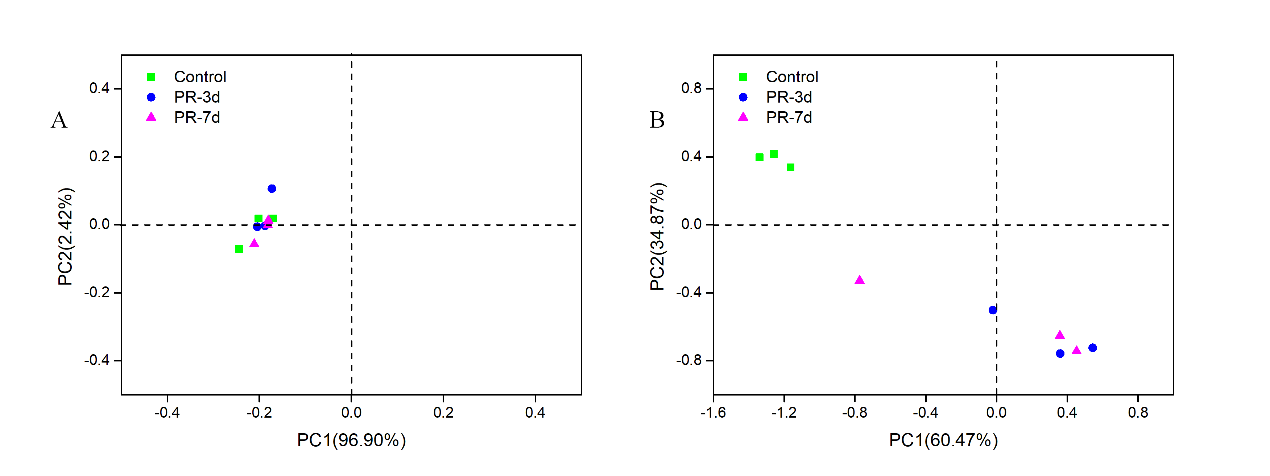


**Figure S4** PCA analysis of tobacco microbial community.

1. Bacteria, (b) Fungus


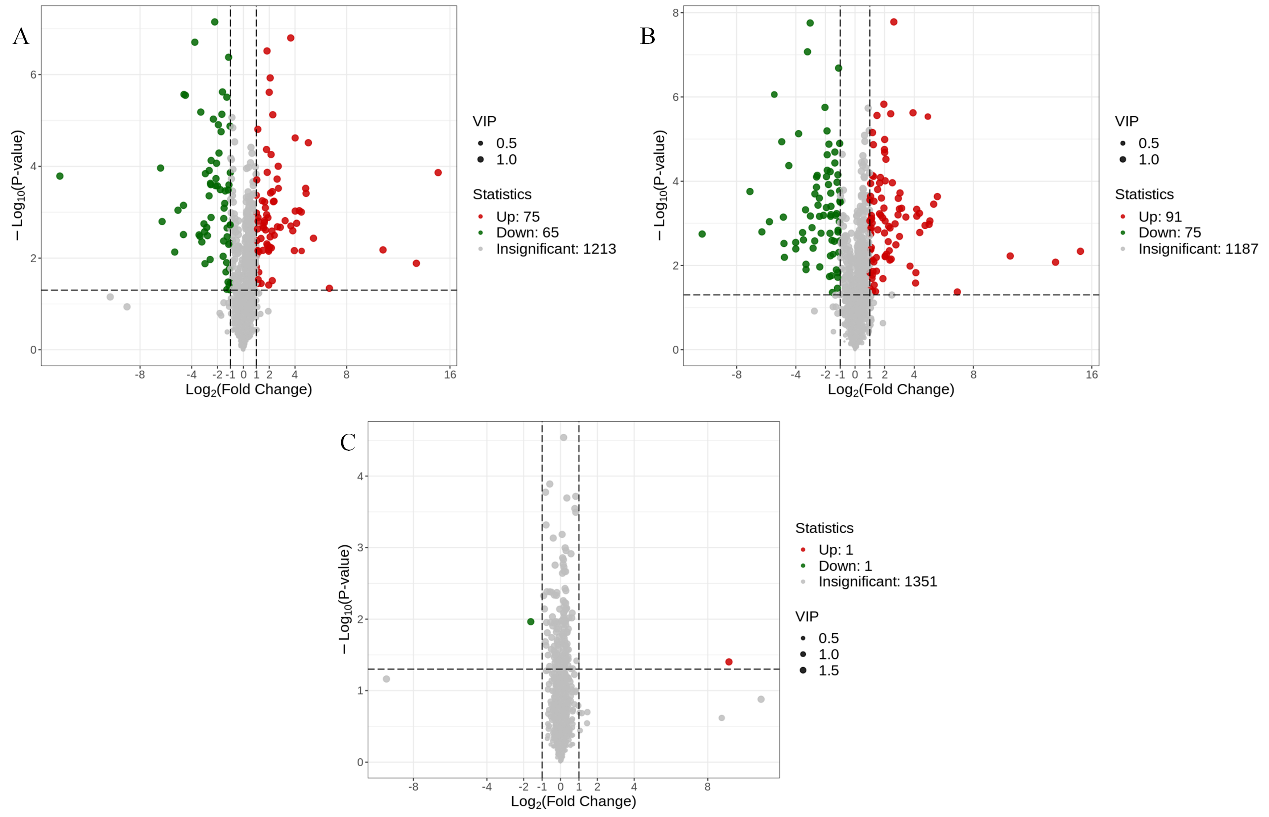


**Figure S5** Volcano diagram of differential metabolites at different fermentation times.
(A) Fermented 3 d VS control, (B) Fermented 7d VS control, (C) Fermented 3 d VS 7 d


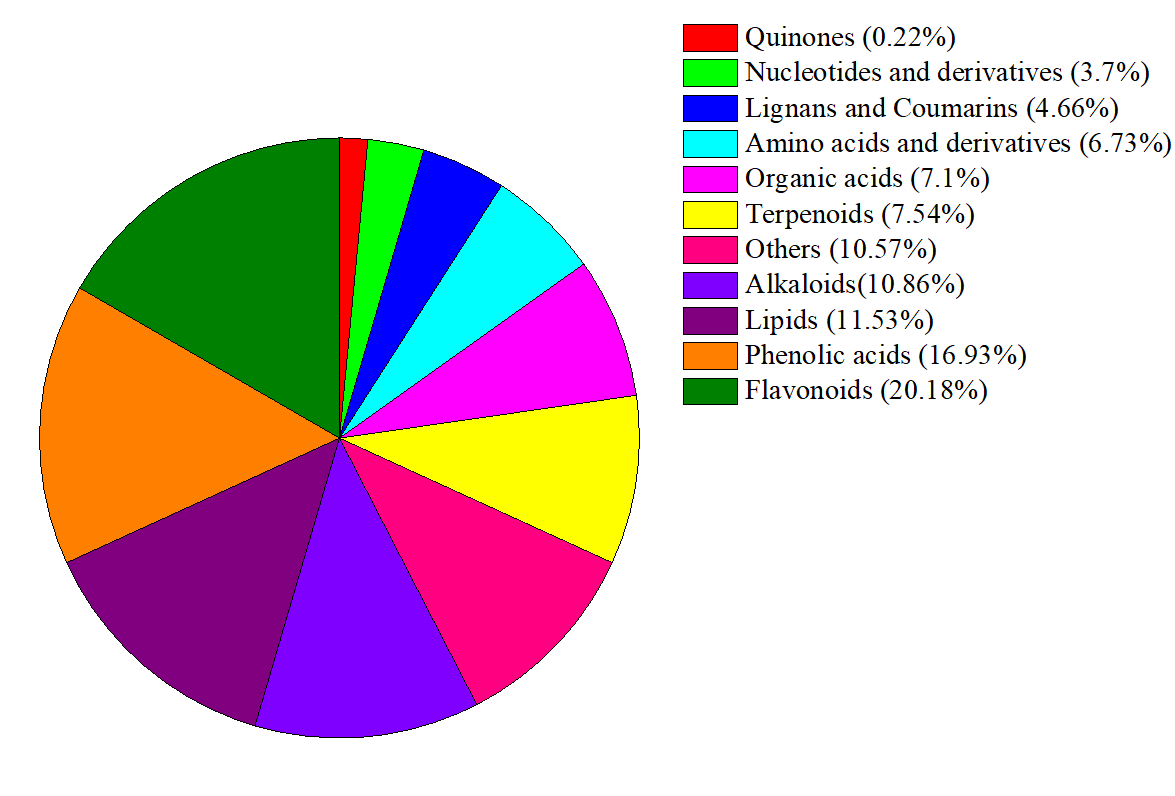


**Figure S6** Proportion of different types of metabolites.


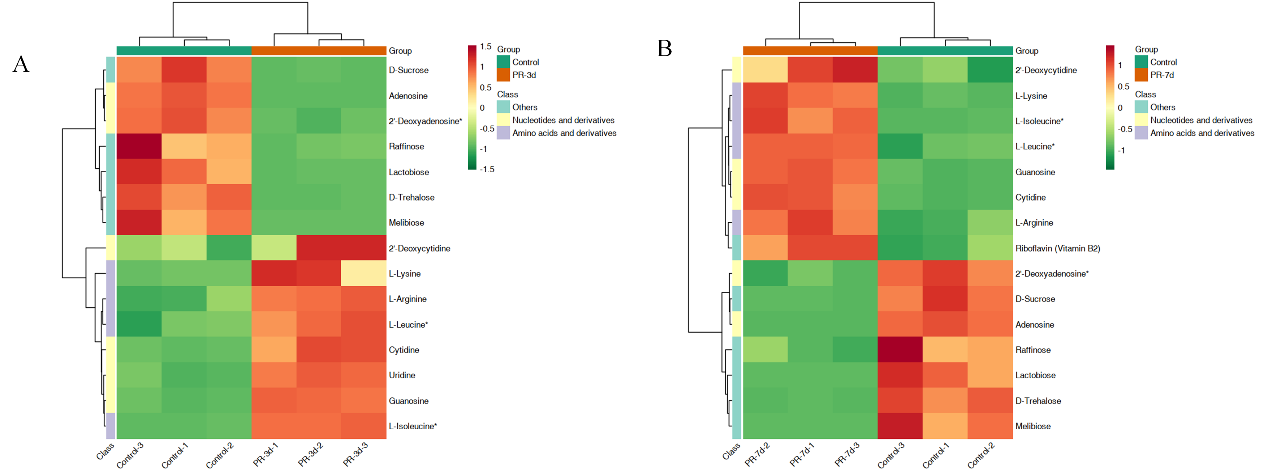


**Figure S7** Cluster analysis of differential metabolites in the ABC transporters pathway.

1. Fermented 3 d, (B) Fermented 7 d


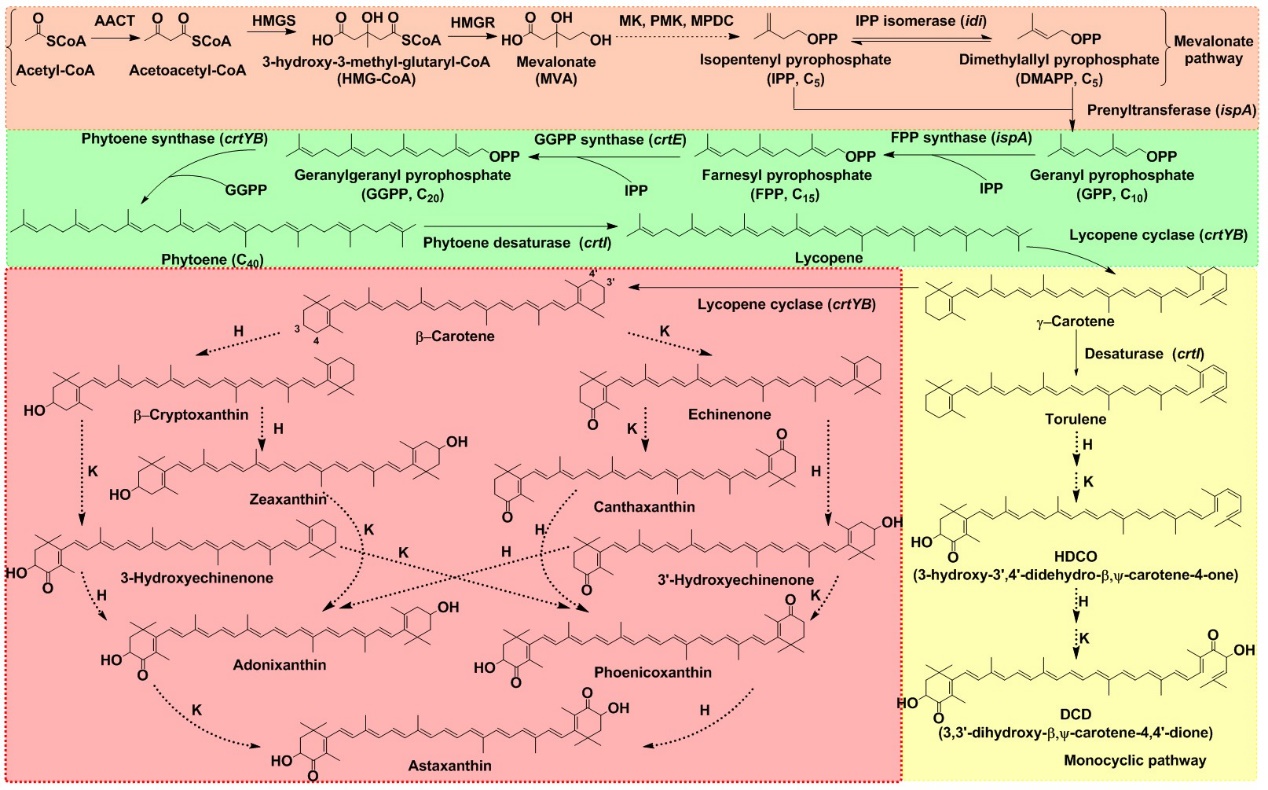


Figure S8 Biosynthetic pathway of carotenoids in *P. rhodozyma*
